# Narcissism on interpersonal circumplex model: Reactions to imaginary abandonment and rejection

**DOI:** 10.3389/fpsyg.2022.987038

**Published:** 2022-12-01

**Authors:** Gamze Şen-Pakyürek, Elif Barışkın

**Affiliations:** ^1^Department of Clinical Psychology, Manisa Celal Bayar University, Manisa, Turkey; ^2^Department of Clinical Psychology, Hacettepe University, Ankara, Turkey; ^3^Department of Psychology, TED University, Ankara, Turkey

**Keywords:** grandiose narcissism, vulnerable narcissism, abandonment, rejection, interpersonal circumplex model

## Abstract

**Aim:**

Narcissism is a direct result of the lack of self-expression. Thus, this trait is enshrined in cycles of strategies to protect self-worth in interpersonal relationships. The aim of the present study was to understand in what way narcissistic individuals understand their interactions with themselves and others.

**Method:**

The study included two groups comprising high grandiose narcissism (GN, *n* = 43) and high vulnerable narcissism (VN, *n* = 44). The participants who received scores that were one standard deviation (SD) above the mean, either on the PNI-grandiosity or on the PNI-vulnerable dimensions, constituted the GN and VN groups among 518 students (Age: *M* = 23.57, *Sd* = 2.13).

**Results:**

The chi-square test was performed to evaluate the dimensions of narcissism with respect to responses of the participants and their partners (behavioral, cognitive, and emotional). The Pathological Narcissism Inventory (PNI) was administered to evaluate narcissistic characteristics. The “Criticism Story” of the Story Completion Inventory in Romantic Relationships (SCIRR) was used for criticism. The circular pattern between the responses of the participants and their partners was examined using the Interpersonal Schemas Scale (ISS). The results revealed that the vulnerable group gave more complementary responses emotionally and made more complementary predictions in terms of the expected reactions from the romantic partner than the grandiose group.

**Discussion:**

The results were discussed in reference to the basic self-esteem-protecting motivations of the groups.

## Introduction

**Figure 1:**
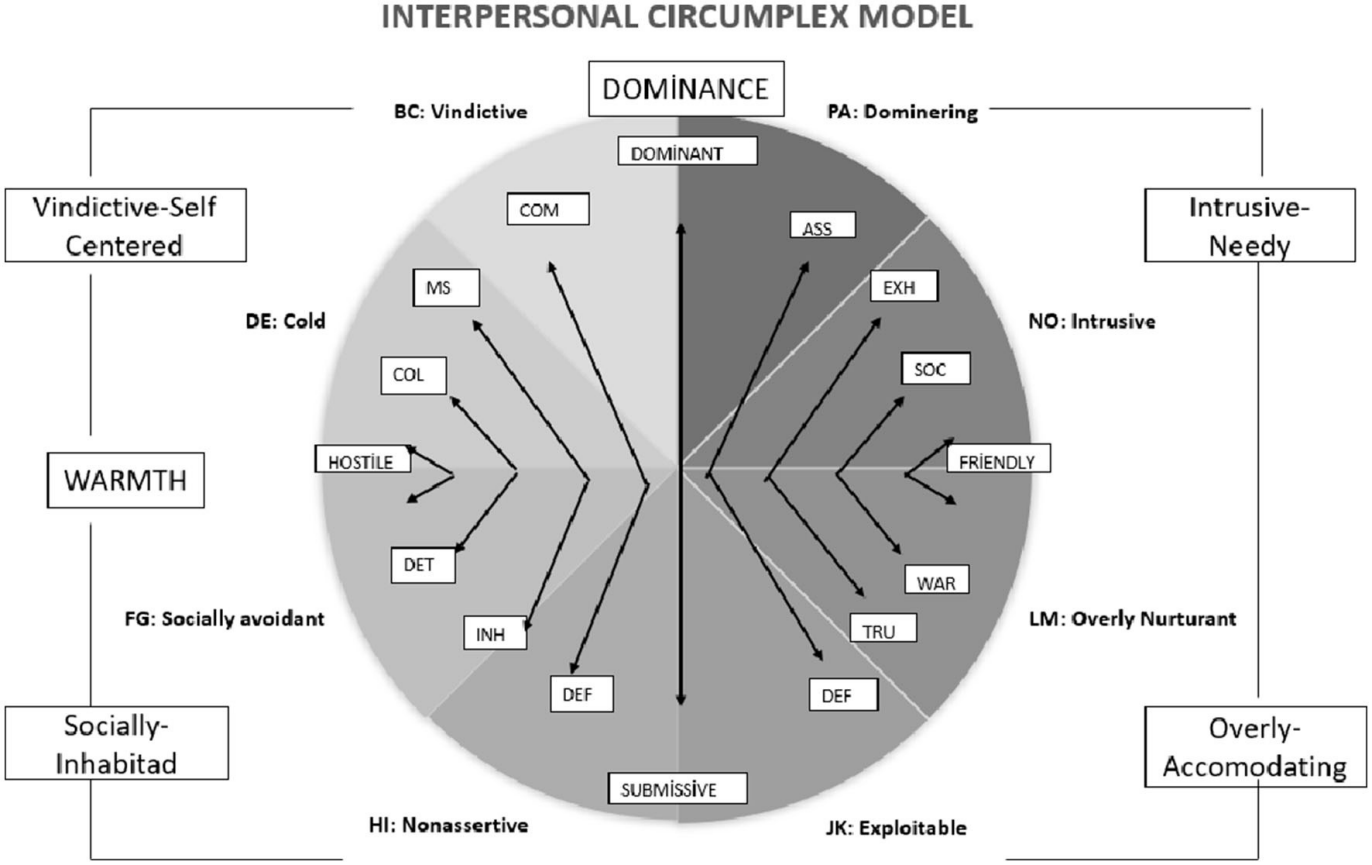
Completion pattern of Kiesler's (1983) interpersonal circumplex model of personality. According to the principle of completion in interpersonal relationships, it enables the emergence of anti-behavior behaviors close to the dimension of dominance-submissive; behaviors close to the size of friendly-hostile lead to the emergence of reactions that will complement them. The polar loop is the interpersonal loop proposed by Kiesler (1983). It forms the basis of the principle of completion of interpersonal relationship patterns. According to the complementarity principle, behaviors in the control dimension lead to the opposite behaviors (e.g., dominant-passive), while behaviors in the coexistence dimension lead to the emergence of reactions from their own class that will complement them (e.g., hostile-hostile, friendly-friendly). The interpersonal circumplex (IPC) model classified interpersonal schema and behaviors into eight octants under orthogonal two axes (Dominance and Warmth). Each octant was described as Domineering (PA), Vindictive (BC), Cold (DE), Socially avoidant (FG), Non-assertive (HI), Exploitable (JK), Overly Nurturant (LM), and Intrusive (NO) characteristics (Horowitz, 2004; Gurtman, 2020).

The Interpersonal Circular Model (IPC, see Figure 1), which is based on the interpersonal personality theory, explains interpersonal behavior with two basic dimensions called “dominance and warmth” (Leary, 1957; Wiggins, 2003). Every point in the IPC space represents a region that is formed by intersection of the two dimensions (reflecting status/power, and friendliness/warmth, respectively). IPC provides the basis for resolving interpersonal dynamics including narcissism (Gurtman, 1992). Early studies on narcissism have generally focused on the grandiose dimension. Although it is conventional to use IPC to understand different personality characteristics, vulnerable narcissism (VN) has received little attention in this aspect. With its IPC components and outcomes, VN has been useful for the examination of the narcissistic personality characteristics (Dickinson and Pincus, 2003; Miller et al., 2007, 2012; Ogrodniczuk et al., 2009; Pincus et al., 2009). Pincus et al. (2009) showed that the subscales of the vulnerable dimension of the Pathological Narcissism Inventory (PNI) fall into three different quartiles: the Vindictive quadrant (high dominant and low warmth), the Avoidant quadrant (low dominant and low warmth), and the Exploitable quadrant (low dominant and high warmth). When examining in terms of disruptive events, Miller et al. (2012) mostly related grandiose narcissism (GN) to a particular interpersonal behavior pattern that is best described in the Vindictive quadrant (high dominant and low warmth), while VN was clearly dependent on interpersonal coldness that manifests either in a submissive or in a dominant style. From this point of view, VN and GN have hostile reactions to destructive events in common, but VN (especially dominance) needs to be examined in a more detailed manner.

Indeed, it has been suggested that personality disorders are diagnostic correlations of interpersonal problems that are defined by the cyclical model (Wiggins et al., 1989; Horowitz et al., 2003). Empirical studies have examined the relationship between grandiose and vulnerable dimensions (Wiggins and Pincus, 1989; Sim and Romney, 1990; Morf and Rhodewalt, 1993; Gurtman, 1996; Tracey et al., 1996). These studies have yielded consistent findings, which showed that narcissistic personality characteristics are located on the hostile-dominant quadrant when their placement on the circle is considered. Studies have shown that not only individuals with a pathological level of narcissism but also individuals with a high level of normal narcissism give intense reactions to destructive experiences (Rhodewalt and Morf, 1998; Dickinson and Pincus, 2003; Pincus et al., 2009). Based on this approach, it is argued that the narcissistic characteristics of individuals who have a high sensitivity to past challenging life events will be activated when coping with the threat and anxiety they faced during abandonment and rejection.

In the last decade, vulnerable narcissism (VN) came to the fore as a separate dimension in narcissism research, and the nonlinear and multidimensional nature of narcissism has been widely accepted (Jauk and Kaufman, 2018). Many studies have supported the examination of narcissism under two basic dimensions (Dickinson and Pincus, 2003; Pincus and Lukowitsky, 2010; Miller et al., 2011, 2012; Rohmann et al., 2012; Stoeber et al., 2015; Gore and Widiger, 2016). Current literature describes the source of narcissism as a well-deserved state of self-importance, and this state emerges in the form of boasting, arrogance, exposure, and manipulativeness (Krizan and Herlache, 2018). In addition, advocacy, irritability, shame, avoidance, and shyness are evaluated as the characteristics of the vulnerable dimension of narcissism (Ellison et al., 2013). In the literature, it is accepted that vulnerable narcissism (VN) contains introversion, imperfection, anxiety, pessimism, and vulnerability to traumas, while grandiose narcissism (GN) includes extroversion, self-confidence, exhibitionism, the need to be admired, and aggression (Wink, 1991; Kohut, 2011, 2018). The interpersonal strategies of both dimensions seem to have common features, in that they are both cold and hostile (Miller et al., 2012; Casale et al., 2016, 2020; Weiss and Miller, 2018; Loeffler et al., 2020; Weiss and Huppert, 2021). However, even at this level, the dimensions differ in terms of emotional and cognitive needs that motivate behavior (Miller et al., 2010, 2011; Wright et al., 2017). In addition to the four poles of these two axes, when four more poles of combinations of these poles are added further, the IPC is divided into eight octanes that define eight areas of interpersonal behaviors/problems (Kiesler, 1996; Horowitz, 2004; Locke, 2006; Gurtman, 2020).

There is no consensus on the common core of the dimensions of narcissism, despite their seemingly opposite features (Jauk et al., 2017). According to the research conducted by Jauk and Kaufman (2018), the grandiose manifestation of narcissism comes to the forefront with increasing levels of narcissism, but the way this trait is exhibited still differs. This study aims to examine the different reflections of the high levels of narcissistic grandiosity and vulnerability. The identification of the differences is crucially important, because the two dimensions of narcissism are related to internalizing and externalizing symptoms and different behaviors (Miller et al., 2011, 2013), in addition to the difference in the need for clinical facilities (Pincus et al., 2009). Given that narcissism with two dimensions is united by important interpersonal problems, the IPC has been useful for providing a better understanding of the characteristics of narcissism with two dimensions in relation to interpersonal behaviors, especially romantic relationships (Dinić and Jovanović, 2021).

Studies have shown that individuals with a high level of narcissism tend to ignore, belittle, or attack the other person when they receive feedback in the form of social rejection that threatens their self-worth (Bushman and Baumeister, 1998; Bushman et al., 2003; Twenge and Campbell, 2003; Campbell et al., 2004). Narcissistic individuals are also known to worry about experiencing the feeling of abandonment with which they could not cope up in their childhood and in their adult life. When faced with rejection, narcissistic individuals exaggerate their personal romantic stories (Rhodewalt and Eddings, 2002). This might be anti-complementary, due to their increased sensitivity to the threats of criticism or conflict (Morf and Rhodewalt, 1993; Horton and Sedikides, 2009). In conclusion, narcissistic individuals have certain expectations in interpersonal relationships. If these expectations are not met (e.g., when they receive negative feedback about their performance or personality), they become angry (Rhodewalt and Morf, 1998) and use personal and interpersonal strategies to eliminate the effects and regulate emotions (Besser and Priel, 2009). Thus, this study aims to analyze the way narcissistic individuals understand the interactions between themselves and others, their expectations from their strategies, and their way of thinking while pondering their next move in destructive events.

In the present study, we examined both GN and VN in relation to the two measures of IPC in the cases of abandonment and rejection with respect to their own and partner's reactions. The main questions of the study included whether there is a general pattern followed in displaying the differences in the responses of VN and GN in the face of abandonment and rejection; and whether there is a general pattern followed in displaying the reactions of VN and GN and his/her partner in the face of abandonment and rejection. We hypothesized that VN would be negatively correlated with warmth and would have a low correlation with dominance, while GN would be related to high dominance and low warmth and the corresponding quadrants. Additionally, we expected that, at the quadrant level, the romantic partner's reaction would complement VN, while the romantic partner's reaction would not complement GN.

## Method

### Participants

University students were randomly reached by email and were asked to complete the Pathological Narcissism Inventory (PNI) (Pincus et al., 2009). Among the 539 people who participated in this phase of study, 21 people who did not meet the inclusion criteria (such as not filling out the inventory randomly and not leaving the inventory mostly blank) were excluded from the study. The remaining 518 people formed the universe of this study. Based on the PNI, 89 individuals who scored one standard deviation (SD) above the mean on the vulnerable dimension (*M* = 130.06, *Sd* = 19.7) and 83 people (*M* = 49.99, *Sd* = 7.44) who scored one standard deviation (SD) above the mean on the grandiose dimension were invited by email to participate in the study. Among them, 29 people (PNI Total: *M* = 133.38, *Sd* = 15.21) had high scores on both dimensions and so were not included in the study. Even among the students who were invited to participate in the present research study, the total number of students who could not participate in the study due to the following reasons amounted to: 16 for missing or incorrect contact information, nine for graduating and leaving the city, and 2 for being married. Even among the responding students (*n* = 518, 39% male) who scored one standard deviation (SD) above the mean of the grandiose dimension (*n* = 54*, M* = 50.33, *Sd* = 9.89) and vulnerable dimension (*n* = 60, *M* = 128.09, *Sd* = 11.58) of the PNI, 83 students were invited to attend a face-to-face interview. The aim of this study was not to carry out experimental research that allows to draw cause–effect inferences or to calculate a universal parameter that is specific to the variables, but to calculate the sample size in which the probability of type-1 and type-2 errors is controlled *via* G power analysis (Faul et al., 2007, 2009). From this point of view, the study sample was determined to be a minimum of 72 participants by the researcher and the thesis advisor for 95% power at a significance level of 0.05 to detect a low-degree (0.3) correlation, and the sample number was increased by 30% to 83 people to prevent possible data loss. The exclusion criteria included having had a psychiatric diagnosis conducted and being married, considering the content of the stories and outliers to control confounding variables. Finally, the study was carried out with two groups comprising participants with high grandiose (GN, *n* = 43) and high vulnerable (VN, *n* = 44) narcissism. Forty-six participants (42%) were in a romantic relationship at the time of study and the duration of relationship ranged from 4 to 72 months *(M* = 19.50, *S* = 17.37, range = 4–72 months). Since the data of the study were collected through face-to-face interviews, there are no missing data.

### Measures

#### Demographic information form

The demographic information form is used to learn more about the demographic features of the study participants. The said form includes information about age, gender, marital status, and any ongoing romantic relationship. The exclusion criteria for the study were psychiatric diagnosis and marriage.

#### Pathological narcissism inventory

Pathological Narcissism Inventory (PNI) was developed by Pincus et al. (2009), while the Turkish adaptation studies were carried out by Sen and Barişkin (2019). The scale is of the 6-point Likert-type comprising 52 items. It measures narcissism by two main factors, namely grandiose and vulnerable, and seven subfactors. A six-factor structure was obtained in the Turkish standardization of the scale, and the factors were named “Recognition Expectations (REX), Grandiose Self (GS), Vulnerable Self (VS), Approval Seeking (AS), Grandiose Fantasy (GF), and Self Sacrificing (SS)”. In the original study, Cronbach's alpha value of the scale was 0.95, and the reliability coefficients of the subscales ranged from 0.78 to 0.93. In the Turkish adaptation study, the Cronbach alpha value for the total score was 0.93, the internal consistency coefficients of the subdimensions ranged between 0.58 and 0.92, and the test–retest reliability coefficients ranged between 0.81 and 0.93. The Cronbach alpha value for the grandiose narcissism dimension was 0.83, while it was 0.93 for the vulnerable narcissism dimension. Within the scope of this study, the internal consistency coefficient of the total score was 0.92, while the internal consistency coefficient of the grandiose narcissism subscale was 0.86. Similarly, the internal consistency coefficient of the vulnerable narcissism (VN) subscale was 0.92, while the internal consistency coefficients of the subdimensions ranged from 0.73 to 0.92.

#### Story completion inventory in romantic relationships

The inventory was created in the form of a semi-structured interview within the scope of this study. It contained hypothetical stories about abandonment and rejection. Following these stories, the participants were asked, “*How would you react in this situation? (Behavioral reaction),”* “*What would you think? (Cognitive reaction)*,” “*How would you feel? (Emotional reaction)*,” and finally, “*How the other person would react to you (Expected reaction)*?” The responses of the participants were coded by raters based on Kiesler's (1983) Cycle Model and the Interpersonal Schemas Scale (ISS). The reliability values of the Story Completion Inventory in Romantic Relationships (SCIRR) were calculated using the Interclass Correlation Coefficient (ICC) of the scores of the reviewers and judges. The discriminant validity values were calculated for the validity of the stories. The reliability coefficients were significant and ranged from 0.84 to 0.97 (*p* < 0.05). The discriminant validity and reliability coefficients for all stories and coding were found to be at an acceptable level (*p* < 0.05). The procedure for the stories is described in detail in the Procedure section of the study.

#### Interpersonal schemas scale

The ISS is developed by Hill and Safran (1994) and aims to evaluate the reactions that people expect from their significant others. The scale reveals what reactions people expect from their significant others in which situations, and how desirable these reactions or interpersonal situations are for people. During the application of the scale, the participants are asked to imagine portraying a behavior for 16 scenarios in the polar cycle model, which is represented in Kiesler's (1983) “1982 Interpersonal Circle” (1982 Interpersonal Circle). Then, they were asked to indicate how they expect other people to behave in the face of these behaviors with respect to eight responses given to them. Standardization studies into Turkish were done by Boyacioglu and Savaşir (1995). Reliability and validity indicators were found to be sufficient. In our study, the reactions to the stories of abandonment and rejection were coded by judges using eight reactions as criteria (e.g., From “A- Takes responsibility or tries to influence” to “H- Shows interest or openly says what she/he thinks.”).

### Procedure

The study was approved by the Ethical Committee of Hacettepe University (No.: 35853172/433-646), Ankara, Turkey and conducted in the Clinical Psychology Research Laboratory of the same university. Participation was on a voluntary basis. Students who scored one standard deviation (SD) above the mean of the vulnerable and/or grandiose dimensions of the PNI were invited by email to attend face-to-face interviews. Then, they were given an informed consent form and a demographic information form, which was followed by the abandonment and rejection story. In the present study, participants were asked to imagine the situation that was described in the story (see Appendix). Then, the following questions were asked: “How would you react in this situation?”, “What would you think?”, “How would you feel?” and finally, “How your partner would react in response to you?”. The questions were asked so as to detect behavioral, cognitive, and emotional aspects of one's own reactions and their partner's expected reaction in return. Interviews were conducted by the researcher and took almost 10–15 min for each participant.

The responses of the participants were recorded. Three judges (a clinical psychology doctoral candidate and a research assistant) classified the responses of the individuals and their partners. Responses were coded by the raters based on the Kiesler (1983) IPC Model and the Interpersonal Schemas Scale. At the end of the procedures, the judges were asked to classify the participants' way of completing the story in one of the eight interpersonal response dimensions of the Interpersonal Schemas Scale (ISS) (e.g., from “A- Takes responsibility or tries to influence” to “H- Shows interest or openly says what she/he thinks.”). The judges coding the responses from the SCIRR were blind to the narcissism dimension of the participant (i.e., grandiose or vulnerable narcissism).

### Data analysis

IBM SPSS 25 was used for conducting statistical analyses. After the development of SCIRR, when examining validity and reliability values, coding and scoring were taken as the basis, and interrater coefficients were calculated using the SPSS program. The judges coding the responses from the SCIRR were blind to the narcissism dimension of the participant (i.e., grandiose or vulnerable narcissism). Then, the Spearman correlation test was used to examine the relationship between their reactions (behavioral, cognitive, and emotional) and their partners' reactions (expected reaction). Finally, a classification was done to understand the pattern between an individual's own reactions and the reactions of their partner based on the Interpersonal Schemas Scale (ISS).

Before the analysis, data entries were checked, missing and extreme values were scanned, and the assumptions were tested. The analyses revealed that the assumptions of normality and variance homogeneity were not met. During this stage of study, the participants consisted of individuals who were one standard deviation (SD) above the mean in terms of pathological narcissism measurement. Thus, the personality characteristics of the sample were not suitable for the normality assumption considering the purpose of the study. In addition, in the non-pathological sample of 518 people, the number of people with one standard deviation (SD) above is not sufficient to carry out parametric tests. Thus, non-parametric tests were employed. The chi-square test was used to examine the relationship between the outcome variable and the input variable. The Mann–Whitney *U-*test was used for pairwise comparisons, which is a non-parametric test to examine whether quantitative-scale observations come from the same distribution. Accordingly, in our study, the Mann–Whitney *U*-test was used to compare vulnerable and grandiose groups. The responses of the participants for themselves and their partners were divided into four complementary levels (high completion, low unrelated, high unrelated, and anti-complementary) to make an evaluation indicating the existence of a pattern between the reactions of the person themselves and the reactions of their partners.

## Results

During this stage, first, the independent sample *t*-test was used to examine the VN and GN groups in terms of age, which revealed no significant differences between the groups (*M*_*female*_ = 25.64, *M*_*male*_ = 23.54). Second, the chi-square test was performed to examine the differences between the groups in terms of gender, relationship status, and reactions to their own and their partner's reactions. The test revealed no significant difference between the GN and VN groups in terms of gender (29% male) and having a romantic relationship (41% Yes; respectively, *X*^2^ = 1.44, *Sd* = 1, *p* = 0.7; *X*^2^ = 0.44, *Sd* = 1, *p* = 0.7). Then, the Kruskal–Wallis-H test was used to evaluate the dimensions of narcissism (grandiose and vulnerable) in the cases of abandonment and rejection. The Mann–Whitney U-test was applied for paired comparisons to determine which narcissism groups caused a significant difference after the Kruskal–Wallis-H test (see Table 1).

**Table 1:** Comparison of different levels of narcissism in terms of demographic characteristics.

			**Grandiose**	**Vulnerable**				
			***N* = 43**	***N* = 44**				
			**Mdn**	**Mdn**	***X* ^2^**	**Df**	**Sig**	**μ**
Abandonment	Self-reaction	1. Behavioral	54,72	53,38	1.57	1	0.58	0.8
		2. Cognitive	50,80	51,73	8.75	1	0.01	0.9
		3. Emotional	52,78	54,51	2.13	1	0.35	0.7
	Romantic partners'	4. Expected	55,98	55,39	0.22	1	0.98	0.6
Rejection	Self-reaction	1. Behavioral	62,09	51,92	3.41	1	0.18	0.7
		2. Cognitive	55,29	55,95	0.02	1	0.99	0.6
		3. Emotional	49,35	63,06	8.17	1	0.02	0.9
	Romantic partners'	4. Expected	47,26	63,81	6,13	1	0.05	0.9

For abandonment, the GN and VN significantly differed in terms of cognitive reactions (*X*^2^ = 8,75, *Sd* = 1, *p* = 0.01). GN (*Mdn* = 50,81) and VN (*Mdn* = 51,83) were on the friendly dominant quadrant, and both had reacted in a low unrelated and anti-complementary manner. This indicates that although the abandonment situation is cold (Quadrant: Hostile-dominant), the GN and VN groups will be “social” (Quadrant: Friendly dominant) and anti-complementary in the case of abandonment, but VN gives more complementary responses than GN.

For rejection, the vulnerable group (*Mdn* = 60.13) gave more complementary responses at the emotional level than the grandiose group (*Mdn* = 49.35; *X*^2^ = 8.17, *Sd* = 1, *p* = 0.02). The reactions of both groups were cold, on the hostile-dominant quadrant, and high complementary, but the VN group was significantly more complementary than the GN (*p* < 0.01). This indicates that although the situation is “distant” (Quadrant: Hostile-submissive), the reactions of both groups will be “cold” (Quadrant: Hostile-dominant) and complementary in the case of rejection, but the VN group emotionally scored significantly higher than the GN group. According to the results of the Mann–Whitney *U*-test to compare the groups in terms of their expected reactions in the case of rejection, the VN (*Mdn* = 63.81) gave more complementary responses than the GN (*Mdn* = 46.26) was found (*U* = 653.00, *p* = 0.00). This indicates that the VN group expect their partner's reaction to be “detached” (Quadrant: Hostile-submissive) and high complementary, while the GN group expect their partner's reactions to be “sociable” (Quadrant: friendly dominant) and high unrelated.

Second, the relationship between one's own reaction (cognitive, emotional, and behavioral) and other's response for both groups was evaluated using the Spearman correlation (see, Table 2). In the case of abandonment, there is a positive correlation between behaviors and cognitions in the GN group with a value of 0.38 (*p* < 0.05) and there is a negative correlation between behaviors and cognitions in the VN group with a value of −0.28 (*p* < 0.05). In the case of rejection, cognitive and behavioral reactions of the person had a significant relationship both for the GN group with a value of 0.53 (*p* < 0.05) and for the VN group with a value of 0.51 (*p* < 0.01). The emotional and cognitive reactions had a significant relationship both in the GN group (*r* = 53, *p* < 0.05) and VN group (*r* = 0.40, *p* < 0.01). In the case of rejection, there is a negative relationship between the partner's expected responses and their own behavioral responses in the GN group (*r* = −0.40, *p* < 0.05), while there is a positive relationship between the partner's expected responses and their own cognitive responses in the VN group *(r* = 0.34, *p* < 0.05). These findings indicate that at high levels of narcissism (for both groups), cognitive and behavioral processes were related to one's own reactions to abandonment while emotional processes were less related or unrelated.

**Table 2:** Correlations between self and others' reactions in return for grandiose and vulnerable narcissism.

			**Grandiose**					**Vulnerable**				
			**1**	**2**	**3**	**4**	**5**	**1**	**2**	**3**	**4**	**5**
Abandonment	Self-reaction	1. Behavioral	–					–				
		2. Cognitive	0.38^*^	–				−0.28^*^	–			
		3. Emotional	−0.15	0.14	–			−10	−0.18	–		
	Romantic partners'	4. Expected	0.09	0.28	−0.12	–		0.00	−0.14	0.13	–	
Rejection	Self-reaction	1. Behavioral	–					–				
		2. Cognitive	0.53^*^	–				0.51^**^	–			
		3. Emotional	0.37^*^	0.53^*^	–			0.31	0.40^**^	–		
	Romantic partners'	4. Expected	−0.40^*^	−0.19	−0.10	–		0.17	0.34^*^	0.14	–	

* p < 0.05,

** p < 0.01.

**Figure 2:**
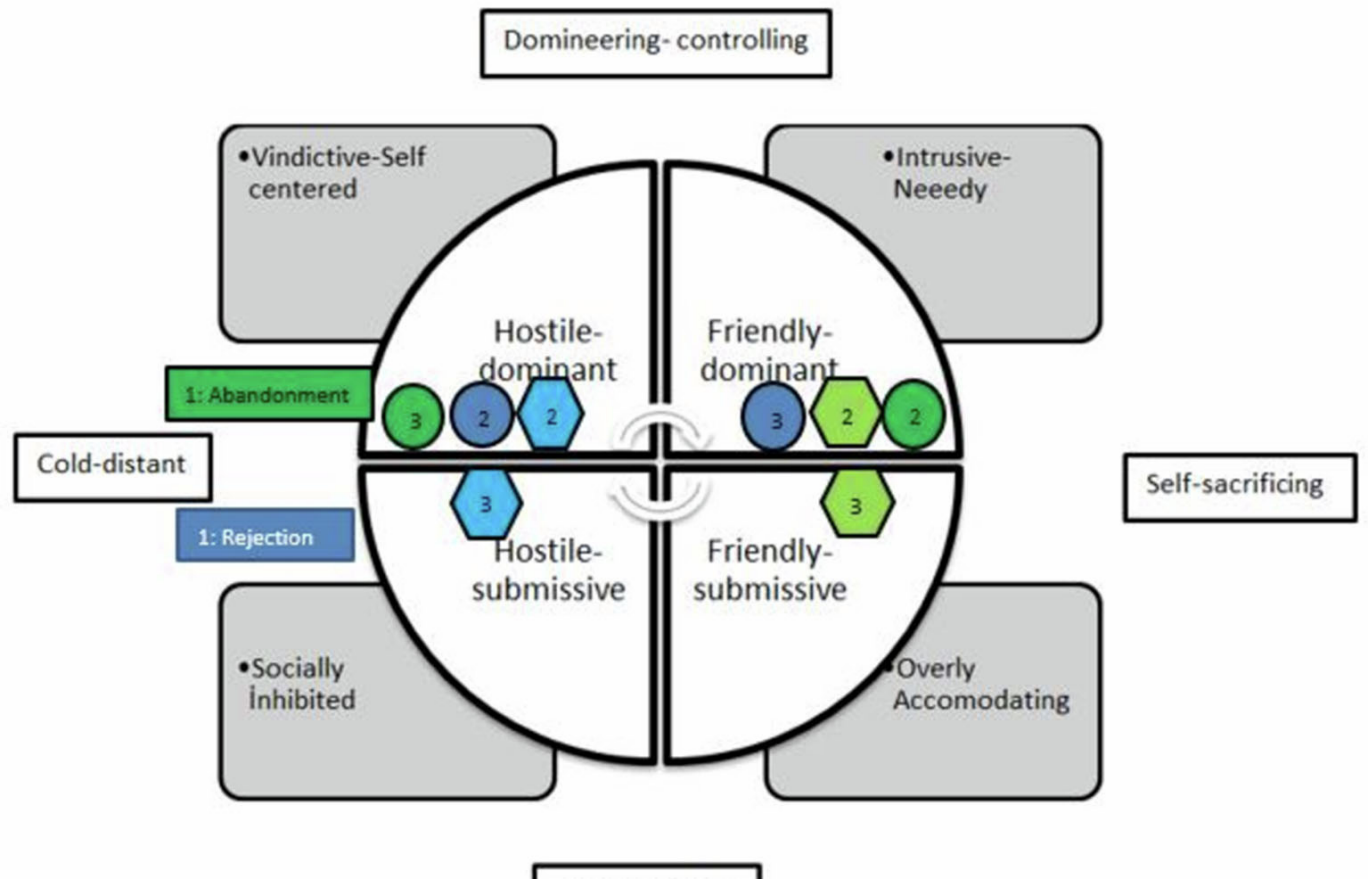
Participants own and romantic partners reactions according to different groups of narcissism in the Kiesler's Circle Model (1983). 1: Place of the stories on Kiesler's circle, 2: own reaction, and 3: expected reaction. Abandonment: Green, Rejection: Blue, Light colors: Vulnerable, Dark colors : Grandiose.

We were interested in examining the circular structure of the IPC, as it varied across different dimensions of narcissism. In the coding phase, the interpersonal situations that the judges determined as “cold” for abandonment and “distant” for rejection were taken as the starting point. Two stories seem to be close to the hostile segment, which is appraised to be the cold-distant octant (hostile-dominant and hostile-submissive, respectively). After determining the starting point for each story, three different reaction points comprising emotions, cognitions, and behaviors for an individual's own reactions were created. Then, all examinations were completed, and the segments of the reactions were determined with respect to the discriminant analysis. According to the results of the discriminant analysis, different dimensions of narcissism were significantly divided, which is shown in Table 3. Figure 2 also summarizes the complementarity results of the reactions.

**Table 3:** Reactions of individuals to compelling interpersonal experiences according to the dimensions of narcissism (GN and VN) and the reactions they expected from their partners and schema compliance.

**Stories**	**Groups**	**Own reactions**	**Quadrant**	**Compliance**	**Schema compliance**	**Expected reactions**	**Quadrant**	**Compliance**	**Schema compliance**
**Abandonment** (**reaction**: cold) (**quadrant:** hostile-dominant)	GN	Sociable	Friendly-dominant	AC	No	Cold	Hostile-dominant	LU	No
	VN	Sociable	Friendly-dominant	AC	No	Warm	Friendly-submissive	HC	Yes
**Rejection** (reaction: distant) (**quadrant**: hostile-submissive)	GN	Cold	Hostile-dominant	HC	Yes	Sociable	Friendly-dominant	HU	No
	VN	Cold	Hostile-dominant	HC	Yes	Detached	Hostile-submissive	HC	Yes

GN, Grandiose narcissism; VN, Vulnerable Narcissism; HC, high complementary; LU, low unrelated; HU, high unrelated; AC, anti complementary.

## Discussion

According to the ego systems model and the interpersonal circular model, relationships in narcissism are mutually dependent on each other through a feedback loop. Thus, an element in the system (cognitive, emotional, or behavioral) activates others, and the narcissistic system becomes active (Foster and Brennan, 2011). In other words, narcissism appears to rise and fall in different ways for the individual at different times (Giacomin and Jordan, 2018). Based on this approach, it can be argued that the narcissistic characteristics of individuals with high sensitivity to past challenging life events become active when coping with the threat and anxiety they face in the cases of abandonment and rejection (Bushman and Baumeister, 1998; Besser and Priel, 2010).

The results of this study revealed that the participants' “own” reactions would be mostly complementary interpersonal reactions, according to different dimensions of narcissism when faced with abandonment and rejection. There were significant differences between the VN and GN in terms of reaction, but there were no self-consistent patterns among their reactions, because they were in different dimensions of narcissism. The vulnerable group gave more complementary responses than the grandiose narcissism group on emotional and cognitive responses and made more complementary predictions in terms of expected reactions from a romantic partner when compared to the grandiose group. A predictable and self-consistent pattern was not found for interpersonal reactions with respect to narcissism dimensions, but important findings were obtained for each theme separately. In the cases of both themes (abandonment and rejection), at high levels of vulnerable narcissism, cognitive and behavioral processes were related to the determination of one's own reactions while emotional processes were less related or unrelated.

According to Pincus and Lukowitsky (2010), vulnerable narcissism was positively associated with high levels of interpersonal sensitivity and reactivity in the case of abandonment. Similar associations were observed for self-absorption/self-admiration and exploitation/entitlement for grandiose narcissism (Zeigler-Hill et al., 2011; Lunbeck, 2020). The abandonment story in this study involves the abandonment of the participants by their partners during an online chat. The reason behind the abandonment was that the partner wanted to accept a job offer in the place where he/she lived for a while. According to the results, there was no cognitive, emotional, or behavioral difference between the VN and GN individuals in terms of their own reactions. Studies have shown that VN individuals are less equipped for self-esteem and using self-development strategies, and they often need feedback from others to manage their self-esteem (Dickinson and Pincus, 2003; Cain et al., 2008; Besser and Priel, 2010; Rohmann et al., 2012). Therefore, individuals with vulnerable narcissism have experienced much more anxiety in the development of relationships with others, are sensitive to signs of abandonment (Leary et al., 1995), and may tend to be more unequipped about abandonment due to the fragile nature of their self-esteem (Mikulincer et al., 1990; Besser and Priel, 2010). Kernberg (1970, 1975) defines inflated self-representation, which is the most prominent feature of grandiose narcissism, as a defense mechanism that can protect oneself against abandonment. Accordingly, individuals may be inclined to deliver the message that they are not affected by abandonment and do not care about abandonment to protect themselves from being abandoned or to protect themselves when faced with abandonment. Emotions might be suppressed, and their cognitions become active, so that the autonomy and self-efficacy that they do not have are not apparent to others and they are not hurt (Young et al., 2006; Besser and Priel, 2009; Brookes, 2015). Individuals with grandiose narcissistic tendencies are known to have a fear of abandonment, see this as a weakness of self-worth and display a defensive grandiosity (Behary and Dieckmann, 2011; Brookes, 2015). Accordingly, although the story of abandonment was coded as cold (Hostile-dominant quadrant), both the GN and VN groups chose to react “sociable” (Friendly dominant quadrant), meaning that they gave anti-complimentary responses. The results revealed that both grandiose and vulnerable individuals thought that they would react sociably, that is, “being close or friendly.” However, one should remember that while there was a positive correlation between the emotions and behaviors of the GN group, the emotions and behaviors of the VN group were negatively correlated when looking at the correlation table. Accordingly, the participants in the grandiose or vulnerable group might prefer not to show their feelings by acting as if there was no problem. Kernberg (1967) stated that as the level of narcissism increases, people feel increasingly unable to cope with being alone and living face-to-face with the constant fear of being abandoned by certain people. A study with clinical samples showed that individuals who were diagnosed with a personality disorder exhibited a more intense and rigid interpersonal behavior pattern than the non-clinical population, and there was a significant difference between their own and others' perceptions (Sim and Romney, 1990). The fact that there is no difference between the groups in terms of the expected reactions from their partners was attributed to the strict nature of the story, which did not allow the participant to negotiate. An examination of the literature reveals that the more compelling the event is, the more likely for individuals to react (Kernis and Sun, 1994). However, when narcissistic individuals react strongly to a life event that threatens their self-esteem, their reaction is stronger than that of an ordinary person (Besser and Priel, 2010; Besser and Zeigler-Hill, 2010; Jauk and Kaufman, 2018). In this respect, it is highly likely that the story of abandonment used in this study might have contained a serious threat to self-esteem for vulnerable and grandiose narcissism, respectively (Gabbard, 1989; Wink, 1991). It is recommended for future researchers to measure, when using the story, how intensely the abandonment situation had been reflected with a Likert-type measurement with a single question.

As regards the situation involving rejection in our study, the individuals in a romantic relationship are rejected by their partner at the time they need social and emotional support, when they demand from their partners, “on the ground that the partner's themself has another plan that participants' him/herself is not invited”. The results of the rejection story show that although the participants would react behaviorally coldly, those in the emotionally vulnerable group feel more intense emotions than the grandiose group. It has been stated that vulnerable narcissistic individuals might experience frustration and anger outbursts if their narcissistic needs are not met by other people, and intense shame follows their anger outbursts (Kohut, 1966, 1977). Individuals with the vulnerable narcissistic personality traits, who are afraid of rejection in interpersonal relations, avoid expressing their feelings of anger openly in their social relations. They experience the emotion quite intensely but implicitly (Rohmann et al., 2012; Bryce et al., 2021; Czarna et al., 2021). This might explain the difference in emotional intensity, although there is no behavioral or cognitive difference (Besser and Priel, 2009). On the other hand, for individuals with grandiose narcissism, anger might be a part of a (distorted) strategy to gain “respect” and retaliate against a person or group who insulted or harmed them (Twenge and Campbell, 2003). Thus, they actively cope with the damage to their self-worth using the externalization mechanism, albeit distorted, and therefore, they experience their emotional reaction less intensely than those in the vulnerable dimension (Miller et al., 2013). This is viewed as a part of the emotional denial defense mechanism or is related to the extent of their perception of rejection.

Due to the nature of narcissism, the individual tends to perceive the reactions of others as compatible with the schema or not to perceive uncertain situations that are not compatible with the schema (Safran, 1990a; Young et al., 2006). The results of the abandonment story revealed that people who scored high on the grandiose dimension thought the other person would react by “being impatient and starting a fight” when they cognitively thought of “showing interest or expressing what they think” after the abandonment experience. The reflections of the grandiose, strong, and not needy self in adult life can come to the fore, especially in emotional relationships. Individuals with the grandiose self-image expect their spouse to continue to act coldly, even if they give moderate, interested, and openly expressive reactions when the relationship is terminated unilaterally by their romantic partner. This expectation could be based on the overcompensatory grandiose coping style that they must acquire as a result of cold and prescriptive parenting styles (Young et al., 2006; Barry et al., 2007). The results also revealed that people who scored high on the vulnerable dimension thought their spouses will react “closely or friendly” when they cognitively think of “showing interest or expressing their thoughts” during the abandonment experience. Although vulnerable individuals may have grandiose fantasies about the self, having a sense of shame toward these thoughts may result in the tendency to undermine their social relationships due to the anxiety of abandonment, loneliness, and/or exclusion in their relationships (Dickinson and Pincus, 2003). This fact revealed that they showed complementary reactions in the opposite direction by choosing “showing interest or expressing their thoughts” in the face of the cold attitude of the spouse in the abandonment story. Afterward, they stated that they expected their spouses to “behave closely or friendly” and showed they expected responses to be in line with the expectation of completion in the face of their own reaction.

The results of the rejection story revealed that people who scored high on the grandiose dimension expected their partners to “show interest and openly express their thoughts” in the case where they “show impatience or start a fight” emotionally. On the other hand, in the case where people who scored high on the vulnerable dimension were emotionally “impatient or fighting”, they expected their partners to “keep away from them and remain indifferent”. Individuals with a high level of grandiose narcissism are expected to respond to their partners with a “cold” attitude in the face of rejection by acting with the grandiose self that they have shaped to protect their self-esteem and psychological wellbeing, which could be triggered by rejection (Pincus et al., 2009). On the other hand, expecting their partners to respond with “social” reactions, even though they gave “cold” reactions, is oppositely complementary, that is, it is high and unrelated. According to the two-sidedness principle emphasized by Safran (1990a) in the interpersonal theory, they selectively perceive the reactions of the other party as “distant” in the face of a “cold” reaction. The grandiose narcissism's intense need for the affirming presence of others causes them to show hypersensitivity to rejection (Stone and Bartholomay, 2020). On the other hand, the fear of being rejected or destroyed may have caused them to close themselves off from negative feedback from their partners and therefore, expect close, warm, and sincere reactions from their partners. For vulnerable narcissism, rejection is experienced quite intensely at the level of interpersonal relations (Besser and Priel, 2010). Individuals with a narcissistic personality structure may exhibit behaviors such as intense and excessive anger in case of rejection (Raskin and Hall, 1979). This finding is based on Ronningstam's (2010) finding that in situations such as abandonment or rejection that threaten self-worth, emotions such as anger and jealousy at the emotional level are clearly expressed by reflecting on the behavior. In vulnerable narcissism, on the other hand, it is consistent with the finding that such feelings are implicitly expressed and not easily expressed. In fact, in this study, individuals who scored high on the vulnerable dimension gave more intense emotional reactions than the grandiose individuals. In addition, it is an expected response with a high level of completion that the vulnerable group expects their partner to respond with “distant” responses when they give “cold” responses. The vulnerable individuals' expected response from the partner in the rejection story is “to stay away and remain indifferent”, which indicates that the partner expects that they would exhibit high-level complementary reactions. This situation is consistent with Safran's (1990b) proposition that, based on the principle of complementary as emphasized in interpersonal theory, they will selectively perceive the other party's reactions as “distant” and accordingly expect a “cold” reaction from the other person. The study of Soygüt et al. (2001) showed that people with antisocial personality characteristics in the same quadrant as narcissism expect similar behaviors from others when they behave in a friendly manner. As the starting point for rejection, the high score descriptions in distant (Quadrant: Hostile-submissive) reactions indicate an inability to express affection to another person, difficulty in making long-term commitments to others, and an inability to be generous, and get along with and forgive others. Thus, the contrast in the expected reaction stems not from the internally similar narcissistic needs of vulnerable and grandiose individuals but from the difference in experiencing those needs, because vulnerable individuals experience these needs implicitly for believing they will not be accepted by their partners (Pincus and Lukowitsky, 2010; Miller et al., 2014).

In summary, although the reactions of individuals in both grandiose and vulnerable dimensions seem to be behaviorally similar, their intensities change. Behind this difference lies the meaning they find in their emotional and cognitive processes. A different pattern in expected reactions from the partner emerges in the evaluation of the partner's reactions, depending on the different dimensions of narcissism in the face of hypothetical stories. The individuals who scored high on the grandiose dimension were self-focused while giving their reactions and evaluating the reactions of their partners while the vulnerable individuals were others-oriented. The basic motivation of both groups is to protect their self-esteem (Zeigler-Hill et al., 2010). For this purpose, the vulnerable individuals drifted away from being the person they were by displaying harmonious behaviors and submissiveness while the grandiose individuals displayed cold and rude behaviors toward other people.

Our study still has some limitations. First, all data in the study are based on self-report measures. In addition, we do not claim that the extreme values refer to the clinical sample, because the PNI version in our country does not include a clinical sample. Therefore, self-reported psychiatric diagnosis is our exclusion criterion. However, psychiatrist's opinion and/or Symptom Checklist (SCL) can be used in future studies that wish to obtain more reliable results in psychiatric diagnosis. Additionally, in such self-report measures, participants might answer questions in a socially desirable way or even exaggerate their positive characteristics. In future studies on narcissism, a measurement tool to control social desirability should be included. Another important limitation of the present study pertains to sample characteristics. Care should be taken while generalizing the findings of this study, because it was conducted with a non-clinical university sample. There is still need for further studies with samples from a wider age group range and with clinical samples, instead of the current non-clinical samples. Moreover, there is a correlation between the two dimensions of PNI, which have a confounding effect. However, we aim to eliminate this effect also by examining grandiose and vulnerable narcissism separately (29 people had high scores on both dimensions and so were not included), using scores that are one standard deviation (1SD) above the mean as the separation point of the two subdimensions, thus highlighting the separate features of the subdimensions.

## Data availability statement

The raw data supporting the conclusions of this article will be made available by the authors, without undue reservation.

## Ethics statement

The studies involving human participants were reviewed and approved by the study was approved by the Ethical Committee of Hacettepe University (no: 35853172/433-646), Ankara, Turkey. The patients/participants provided their written informed consent to participate in this study.

## Author contributions

This article is based on the doctoral dissertation of GŠ-P titled ‘Narcissistic Patterns and Responses to Distressing Interpersonal Experiences: An Investigation on University Sample Based on Cognitive Interpersonal Theory’, which was being conducted at Hacettepe University Department of Clinical Psychology. EB was the Ph.D. adviser for this dissertation. All authors listed have made a substantial, direct, and intellectual contribution to the work and approved it for publication.

## Conflict of interest

The authors declare that the research was conducted in the absence of any commercial or financial relationships that could be construed as a potential conflict of interest.

## Publisher's note

All claims expressed in this article are solely those of the authors and do not necessarily represent those of their affiliated organizations, or those of the publisher, the editors and the reviewers. Any product that may be evaluated in this article, or claim that may be made by its manufacturer, is not guaranteed or endorsed by the publisher.

## Appendix

**Table A1:** Abandonment and rejection stories, validity and reliability.

**Themes**	**Abandonment**		**Rejection**		
Stories	Your partner had to live abroad for a while due to his/her job. You have a relationship for a long time and both of you are happy to be together. One month before her/his return, your partner said she/he should talk to you about an important issue. While talking on the Internet, you asked him/her what the topic he/she wanted to talk to you about. Your partner says: ‘***I received an offer to job here. I intend to accept. We cannot continue our relationship like this; I have decided to break up with you.”***		You are having a hectic day, at the end of this day, you want to relax and plan to spend time with your partner. You need your partners' support. You think it will be good for you to spend time with him/her. Near you finish your work; you call your partner on the phone and offer to go out. Your partner says: **‘Tonight? Absolutely not. I made another plan for myself.”**		
**The discriminant validity of the story among other stories:**					
**Abandonment M (** ***Sd*** **)**					
Reviewer (*n =* 10)		Abandonment: 9,55 (*1,01*)		Rejection: 3,22 (*3,38*)	
Judges n(=3)		Abandonment :9,67 (*0,33*)		Rejection: 3,33 (*1,67*)	
**Rejection M (** ***Sd*** **)**					
Reviewer (*n =* 10)		Abandonment: 1,44 *(0,71*)		Rejection: 9,11 (*1,69*)	
Judges n(=3)		Abandonment :1 *(0*)		Rejection: 8 (*1*)	
			**Kiesler Circle Octant**		
	**Suitability of the stories to the theme**	**The stories**	**Own reactions**	**Partners reactions**	
	Reviewer (*n =* 10)	Judges (*n =* 3)	Inter-rater (*n =* 5)	Inter-rater (*n =* 3)	Inter-rater (*n =* 3)
**Abandonment**	ICC = 0.92 *p* < 0.01	ICC = 0.96, *p* < 0.01	ICC = 0.84, *p* < 0.05	ICC = 0.93, *p* < 0.05	ICC= 0.92, *p* < 0.05
**Rejection**	ICC = 0.96 *p* < 0.01	ICC = 0.94, *p* < 0.01	ICC = 0.86, *p* < 0.05	ICC = 0.87 *p* < 0.05	ICC = 0.93 *p* < 0.05
